# Direct current effects on afferent and hair cell to elicit natural firing patterns

**DOI:** 10.1016/j.isci.2021.102205

**Published:** 2021-02-20

**Authors:** Cynthia R. Steinhardt, Gene Y. Fridman

**Affiliations:** 1 Department of Biomedical Engineering, Johns Hopkins University, Baltimore, MD 21217, USA; 2 Department of Otolaryngology Head and Neck Surgery, Johns Hopkins School of Medicine, Baltimore, MD 21205, USA; 3 Department of Electrical and Computer Engineering, Johns Hopkins University, Baltimore, MD 21217, USA

**Keywords:** Biological Sciences, Cellular Neuroscience, Neuroscience

## Abstract

In contrast to the conventional pulsatile neuromodulation that excites neurons, galvanic or direct current stimulation can excite, inhibit, or sensitize neurons. The vestibular system presents an excellent system for studying galvanic neural interface due to the spontaneously firing afferent activity that needs to be either suppressed or excited to convey head motion sensation. We determine the cellular mechanisms underlying the beneficial properties of galvanic vestibular stimulation (GVS) by creating a computational model of the vestibular end organ that elicits all experimentally observed response characteristics to GVS simultaneously. When GVS was modeled to affect the axon alone, the complete experimental data could not be replicated. We found that if GVS affects hair cell vesicle release and axonal excitability simultaneously, our modeling results matched all experimental observations. We conclude that contrary to the conventional belief that GVS affects only axons, the hair cells are likely also affected by this stimulation paradigm.

## Highlights

• Galvanic vestibular stimulation was shown to evoke naturalistic neural responses
• Conventional understanding maintains that it affects only afferent axons
• In contrast, our work suggests that it affects both hair cells and afferents
• Our work further explains the likely underlying mechanisms of these effects

## Graphical abstract

**Figure:**
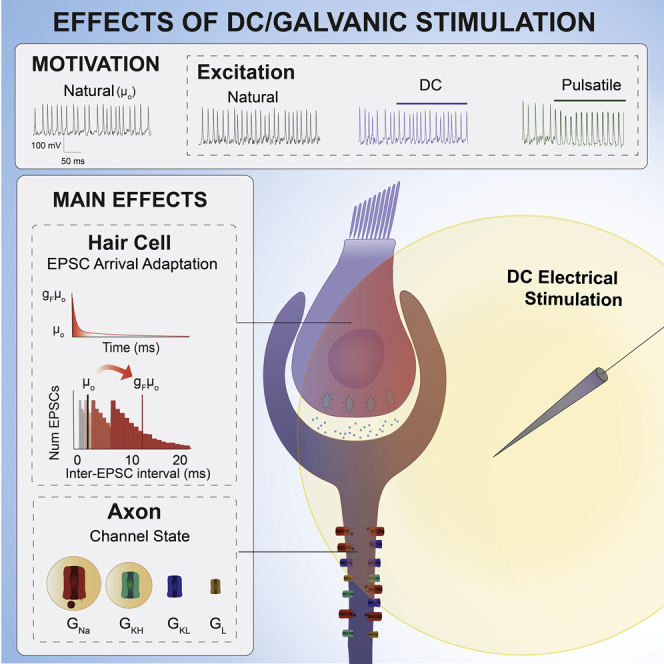

## Introduction

In contrast to conventional pulsatile neural prostheses used to excite neural targets (Loeb, 2018), direct current (DC) neuromodulation emerged as having potential for use in a variety of new medical treatments due to its unique ability to evoke a broad range of beneficial clinical effects on target neurons (Aplin and Fridman, 2019). These have been shown in its ability to achieve peripheral nerve block for pain suppression (Bhadra and Kilgore, 2004; Yang et al., 2018), modulate cortical activity and synaptic connectivity for psychiatric treatments (Bikson et al., 2004; Radman et al., 2007; Brunoni et al., 2012), and excite and inhibit vestibular afferent activity to treat balance disorders (Manca et al., 2019; Aplin et al., 2020). Recent innovations with DC stimulation technology have also led to the development of safe direct current stimulation (SDCS) (Fridman and Della Santina, 2013; Cheng et al., 2017; Fridman, 2017; Ou and Fridman, 2017; Aplin and Fridman, 2019), which makes it possible to chronically deliver localized direct ionic current from an implantable device. Preliminary behavioral testing of the SDCS for vestibular balance disorders as well as for the treatment of pain suppression revealed that DC neuromodulation has multiple beneficial effects on targeted neural populations that cannot be produced with pulsatile stimulation, including inhibiting, exciting, and sensitizing neural targets in a natural, desynchronized manner (Yang et al., 2018; Aplin and Fridman, 2019; Aplin et al., 2019a, 2019b). Although these behavioral results are encouraging, the cellular mechanisms that respond to electric fields are not well understood. The term “DC” is used in neuromodulation to mean a continually delivered current in contrast to pulses. To be consistent with the terminology used in the field of vestibular neuromodulation that is addressed here specifically, we refer to this non-pulsatile current delivery as “galvanic vestibular stimulus” or GVS.

In the vestibular system, GVS has been used in invasive and non-invasive studies much earlier than in other systems, likely because it can be activated from external electrodes (Purkyne, 1819; Fitzpatrick and Day, 2004). For this reason, several studies of single neuron responses to GVS exist in vestibular afferents (Goldberg et al., 1984; Kim et al., 2011; Gensberger et al., 2016; Kwan et al., 2019; Manca et al., 2019). In the vestibular system, three types of afferents, termed regular, irregular, and dimorphic, each receive inputs from a single to several hair cells. Irregular afferents fire with irregular inter spike intervals (high coefficient of variance (CV)) and receive inputs from type I hair cells via calyx type synapses, regular afferents receive inputs from type II hair cells and fire with higher firing regularity (low CV), and dimorphic afferents receive input from a combination of these two types of hair cells. The hair cells release glutamate into the afferent terminals, which in turn produce excitatory EPSPs mediated by the AMPA receptors (Eatock and Songer, 2011; Kirk et al., 2017). Vestibular afferents fire at a high spontaneous rate, which allows both excitatory and inhibitory effects of stimulation to be analyzed.

**Figure 1:**
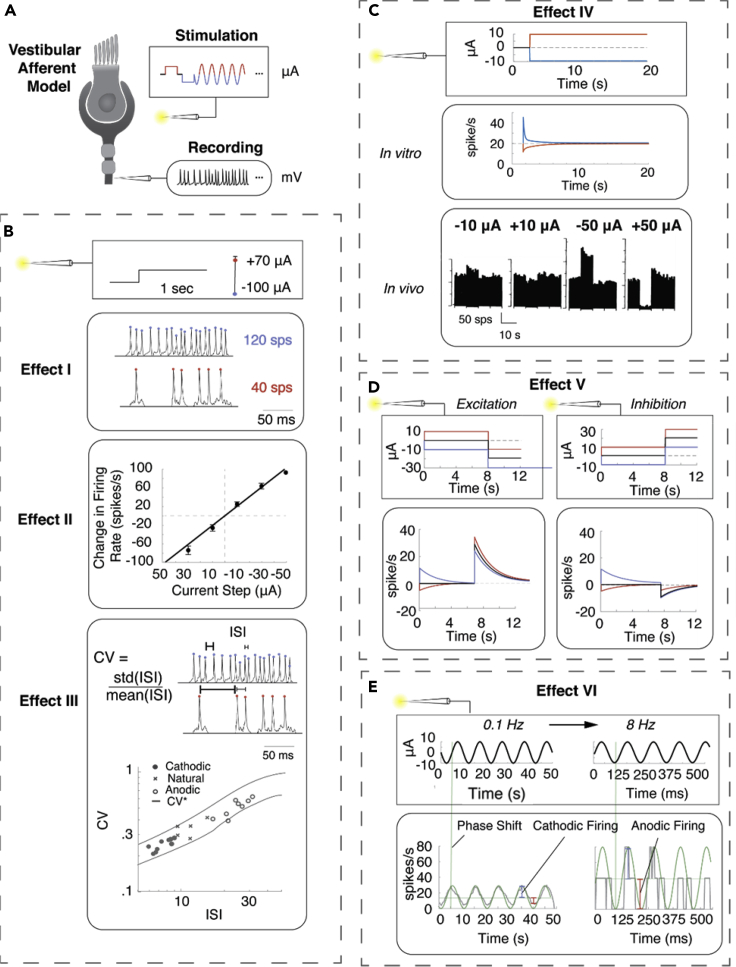
The six distinctive effects of GVS stimulation (A) Square boxes indicate the GVS stimulus, and rounded boxes represent the corresponding neural responses from the vestibular afferent. (B) Transient response patterns. Effect I: low-amplitude GVS stimulation increases and decreases firing rate with cathodic (blue) and anodic (red) current. Effect II: cathodic GVS stimulation can cause dramatic increases in firing rate of up to 2.5 spikes per second (sps) per μA. Data are presented as mean ± std. Effect III: GVS stimulation can maintain firing regularity (CV) while changing firing rate. (C) Long duration adaptation. Effect IV: long-term GVS stimulation induces an immediate change in firing rate that adapts to a new baseline firing rate on the scale of seconds. *In vivo* (black) adaptation occurs with baseline offset in firing rate. (D) Adaptation from different GVS-evoked baselines. Effect V: after a baseline of GVS stimulation, the afferent shows a smaller inhibitory response after and inhibitory (anodic) baseline and smaller excitatory response after an excitatory baseline. (E) Responses to sinusoidal modulation. Effect VI: sinusoidal GVS modulation leads to increased/decreased firing rate in the cathodic/anodic half of a cycle with increased frequency of sine wave, and the neuronal response shows a phase lead for frequencies above 4 Hz that decreases to zero around 4–8 Hz.

GVS modulation in the vestibular system has revealed a number of effects of galvanic stimulation on neurons that require explanation summarized in Figure 1: (I) low amplitude GVS can both increase and decrease firing rate depending on the polarity(Goldberg et al., 1984; Manca et al., 2019); (II) cathodic GVS can cause dramatic increases in firing rate of up to 2.5 spikes per second (sps) per μA (Goldberg et al., 1984); (III) vestibular afferents fire with specific spiking regularity or coefficient of variance (CV) profile termed CV∗, and GVS can maintain this regularity while changing firing rate (Goldberg et al., 1984); (IV) long-duration GVS step induces an immediate change in firing rate that adapts back to a new baseline firing rate on the scale of seconds (Goldberg et al., 1984; Manca et al., 2019); (V) after a baseline of GVS, the afferent appears to sensitize, showing a weaker inhibitory response after an inhibitory (anodic) baseline and weaker excitatory response after an excitatory baseline (Manca et al., 2019); (VI) sinusoidal GVS leads to increased/decreased firing rate in the cathodic/anodic half of a cycle with increased frequency of sine wave, and the neuronal response shows a phase lead for frequencies above 4 Hz that decreases to zero around 4–8 Hz (Gensberger et al., 2016; Manca et al., 2019). Together these results create a set of vestibular afferent response characteristics to GVS that are informative about vestibular function and targets of galvanic stimuli.

Based on the range of the galvanic-affected response characteristics that appear to match natural mechanically evoked firing behavior of the afferents (Goldberg et al., 1984; Manca et al., 2019) as well as mechanically evoked vestibulo ocular reflex (VOR) response (Kwan et al., 2019; Aplin et al., 2020), we hypothesize that GVS must be activating natural cellular mechanisms in both the axon of the afferent and the hair cell.

To test this hypothesis, we systematically modified a computational model of a GVS-stimulated axon-hair cell complex until it could completely reproduce all normal and GVS-induced responses. We began this procedure by implementing the Hight and Kalluri (HK) model, the most recent and detailed mechanistic model of the vestibular afferent (Hight and Kalluri, 2016) and subjecting it to electric fields induced by locally applied GVS (within 1mm). This model assumes single hair cell input to an afferent. Selection of EPSC properties determines the regularity of the afferent's firing rate. We first modified model parameters to simulate afferents with firing ranges and statistics of typical *in vivo* neurons. We found this model was unable to reproduce all observed effects of GVS stimulation. Then, we added simulations of physiologically relevant hair cell and synaptic behaviors not previously present in the model, based on our hypothesis. We conclude that all experimentally observed behaviors can be replicated completely when the hair cell and synaptic modulation by GVS are introduced into the model. The results section describes these systematic modeling results and modifications of the necessary axonal and hair cell mechanisms implicated in GVS neuromodulation (summarized in Table 1).

**Table 1:** Summary of findings

Effect number	Experimental observations	Explanation
I	Anodic GVS increases firing rate. Cathodic GVS decreases firing rate.	**Axonal effect**: GVS stimulation affects the axon by increasing the membrane potential with cathodic stimulation. This makes EPSCs of lower amplitude more likely to become APs. Anodic stimulation decreases the membrane potential so that EPSCs just above threshold become too small to induce an AP.
II	Change in irregular afferent's firing rate to GVS amplitude is ~2 spikes/μA.	**Axonal and synaptic NQ effect**: this increase and decrease in stimulation is primarily due to GVS stimulation changing axon reactivity to ESPC inputs. However, this effect must be magnified by the non-quantal effect to produce this large of a change in firing rate with respect to current amplitude change.
III	CV of GVS-evoked APs follows natural progression of CV∗.	**Axonal effect**: GVS stimulation is changing the proportion of EPSCs that become APs. These EPSCs are released at a rate drawn from a single probability distribution as in the normal physiological system in agreement with the concept of sampling variance.
IV	Change in current causes adaptation in firing rate in response to step in GVS.	**Hair cell effect**: this effect has the same time constant and relative magnitude of effect for cathodic and anodic GVS stimulation as for excitatory and inhibitory mechanical stimulation of the hair cell. We conclude that this effect is due to GVS stimulation activating the same pathway in the hair cell that is activated with mechanical stimulation.
V	Steps of GVS introduced after a baseline GVS presentation result in proportionally different response amplitudes that depend on the baseline in the *in vitro* studies but not in the *in vivo* studies.	**Hair cell and axonal effect**: this effect was the result of *in vitro* neurons having a small firing rate such that an anodic baseline lowered baseline firing rate and additional steps of anodic stimulation draw the firing rate toward zero, causing a plateau. Similarly, cathodic stimulation drove baseline firing rate toward the upper limit of the *in vitro* firing rate.
VI	Sinusoidal GVS modulation suggests a high pass filtering effect.	**Hair cell effect**: the hair cell pathway that is related to the adaptation effect acts as a high-pass filtering effect with a cutoff at around 8 Hz.

Observed effects of GVS stimulation on vestibular afferents and the physiological explanation predicted by our model.

## Results

Our approach was to uncover the neural targets of GVS stimulation by determining the necessary features and parameters for a mechanistic vestibular afferent model to produce the known responses to GVS stimulation discovered through electrophysiology experiments on vestibular afferents. We compared how well our model produces GVS effects with experimental results from two studies. These studies use different preparations that result in afferents with distinct firing properties. The *in vivo* study (Goldberg et al., 1984) was performed on squirrel monkey afferents with a broad firing range (0–300 sps) and high spontaneous rate (100–120 sps) using an extracellular electrode positioned 2 mm from afferents in the perilymphatic space of the vestibular labyrinth. The second study was recently performed in our laboratory (Manca et al., 2019). It used an *in vitro* preparation in which afferents respond with a low spontaneous rate (15–20 sps) and a narrow firing range (0–60 sps). These experiments applied GVS through micropipettes to an explanted mouse vestibular crista, while acquiring action potentials using an extracellular loose-patch pipette technique. For all simulations we used the same number of simulated neurons as the neurons from each experiment with the same firing rates and ranges.

Extracellular electric fields are typically assumed to affect axons at significantly lower amplitudes than smaller cells (Smith and Goldberg, 1986; Rattay, 1999; Steinhardt and Fridman, 2020). Therefore, we first determined whether GVS produces all firing effects solely through interactions with the axon.

**Figure 2:**
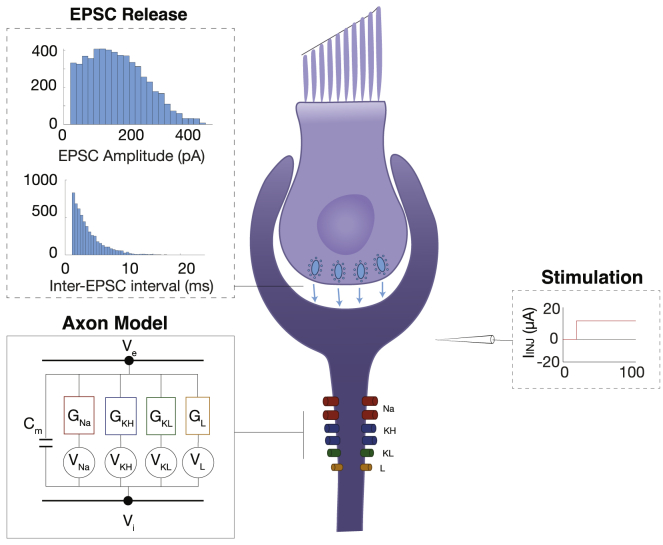
Diagram of the axonal model based on the Hight and Kalluri model

The HK model uses three specific voltage gated channels—a sodium (Na), high-voltage gated potassium (KH), and low-voltage gated potassium (KL) channel—as well as a leak channel to reproduce axon firing dynamics (Figure 2). Spontaneous firing in the axon model is driven by simulated excitatory postsynaptic currents (EPSCs), an axonal manifestation of vesicle release from the hair cell. EPSC arrival is controlled by two stochastic functions: one that determines EPSC amplitude, set with EPSC scaling (*K*), and one that determines EPSC arrival rate by setting the average inter-EPSC interval$\;\left(\mu_{o}\right)$. The HK model suggests that these mechanisms in concert are necessary to generate realistic spontaneous vestibular afferent firing (Figure 2). We first disentangled how each of these mechanisms is contributing to firing through exposure to GVS stimulation.

### Unmodified HK model is insufficient to reproduce GVS-evoked responses

**Figure 3:**
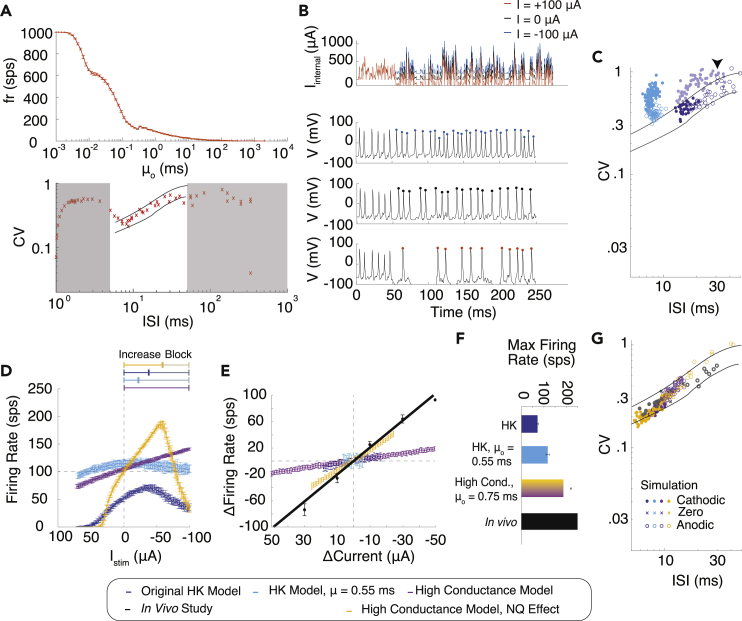
Role of afferent axon in GVS response (A) Changes in mean EPSC arrival rate $\mu_{o}$ result in increased firing rate (top) and conformance to CV∗ (bottom) using original HK model. White region in bottom plot indicates the zone shown in the experimental *in vivo* data with CV∗ boundaries for the irregular neuron. (B) EPSC (top) and membrane voltage V (lower three). GVS is turned on at 50 ms. (top V) Cathodic current increases EPSC baseline bringing the membrane potential closer to depolarization causing more APs (blue). (middle V) No GVS. (bottom V) Anodic current decreases EPSC baseline bringing it closer to hyperpolarization causing fewer APs (orange). (C–G) Dark blue: standard KH model, Light blue: HK model modified with $\mu_{o}$ = 0.55 ms, Purple: HK model with $\mu_{o}$= 0.75 ms and high conductance, Yellow: HK model with $\mu_{o}$ = 0.75 ms, high conductance, and NQ effect, Black: experimental *in vivo* data. (C) CV∗ and CV as GVS current is applied (KH model modified with $\mu_{o}$ = 0.55 ms light blue, standard HK with $\mu_{o}$ = 3 ms dark blue); arrow points to the shaded dark blue points that occurred during the Cathodic Block. Open circles are anodic stimulation; filled circles are cathodic stimulation. (D) Firing rate as a function of GVS stimulation amplitude. (E) Change in firing rate as a function of stimulation amplitude. (F) Maximum firing rates of the responses. (G) CV of the responses. Lines indicate experimental CV∗ from the *in vivo* experiment. All statistics are presented as mean ± std.

The assays for replicating the *in vivo* firing statistics (Effects I–III in Figure 1B) are (1) the mean spontaneous firing rate (fr_o_) of 100–120 sps, (2) change in spike rate in response to GVS steps (dfr/dCurrent ≈ −2 sps/μA) in response to GVS steps between −100 μA and +100 μA, (3) maximum firing rate (fr_max_ > 200 sps) and firing regularity that remains within CV∗ as firing rate changes (Goldberg et al., 1984). These are all depicted in black in Figures 3C–3F.

We first tested the ability of the unmodified HK model to reproduce firing statistics with and without exposure to GVS stimulation. With the original parameters, fr_o_ = 52.7 ± 3.3 sps (N = 19) (Figures 3C–3F dark blue) is significantly below the activity recorded experimentally. When we applied GVS steps at the amplitudes ranging between −100 μA and +100 μA, we observed that although the CV∗ profile was maintained (Figure 3C dark blue), the maximum firing rate was 70.6 ± 6.2 sps in response to −46 μA, significantly lower than that recorded experimentally (>200sps). The slope of the response to GVS (dfr/dCurrent) was −0.5 sps/μΑ (95% confidence interval (CI) [−0.47, −0.55]) compared with the ∼2 sps/μA seen experimentally (Figures 3C–3F dark blue). Increasing the cathodic stimulus beyond −45 μA decreased the axonal response, suggesting cathodic stimulus interference (“Cathodic Block”) (Figure 3D dark blue). This block was previously observed (Bhadra and Kilgore, 2004) and likely is due to GVS inducing exceedingly high membrane potentials at the axon, causing voltage-gated sodium channels to be held in the inactivated state and prevented from reopening.

### EPSC arrival rate increases firing rate without affecting CV∗

As spontaneous firing is induced through changes in membrane potential by EPSCs, we first hypothesized that increasing the rate of EPSC arrival will increase fr_o_ and GVS effects on firing rate at the axon. We examined the effect of changing $\mu_{o}$ in absence of GVS stimulation. We targeted this aspect of EPSCs due to the existing evidence that the release rate is modulated as a function of mechanical motion (Smith and Goldberg, 1986), whereas the amplitudes of the EPSCs (K) remain nearly constant (Dulon et al., 2009). This phenomenon is likely due to vestibular afferents having specialized ribbon synapses and multivesicular release that allows sub millisecond EPSC rate changes to hair cell motion (Grant et al., 2010; Eatock and Songer, 2011). Varying $\mu_{o}$ from 0.1 ms to 250 ms produced firing rates of up to 1ksps, while maintaining the appropriate CV∗ behavior (Figure 3A). We found that faster EPSC arrival is more likely to generate larger changes in membrane potential more quickly, producing larger firing rates.

To match the normal *in vivo* spontaneous activity, we then decreased $\mu_{o}$ to 0.75 ms to achieve fr_o_ = 102.8 ± 3.7 sps. However, with this change to $\mu_{o}$, the model produced responses that were significantly different than those observed experimentally in response to GVS stimulation. Cathodic and anodic stimulation both decreased firing rate within 50 μA, resulting in a slope of −0.04 sps/μΑ, CI [−0.2, 0.29] (Figure 3D light blue). The maximum induced firing rate reached only 120 sps, and the CV∗ of the spiking activity was no longer maintained (Figure 3C light blue). This suggests that the axonal response to GVS stimulation interferes with the EPSC response that would occur naturally.

### Channel conductances determine maximum firing rate and firing regularity in response to GVS

GVS steps create a baseline change in membrane potential that shifts all EPSCs at the membrane uniformly in a depolarizing or hyperpolarizing direction (Figure 3B dark blue and red, respectively). The positive shift in EPSC height with cathodic stimulation raise the previously slightly subthreshold EPSCs above the firing threshold, increasing the number of action potentials (APs). The anodic baseline shift lowers the height of EPSCs that would normally raise the membrane potential high enough to reach AP firing threshold, reducing the firing rate. When $\mu_{o}$ was set to 3 ms as indicated by the HK model, in the range of cathodic stimulation that produces Cathodic Block (−45 μΑ to −100 μΑ), Na channels had a lower probability of opening in response to increases in membrane potential with EPSCs (Figure 3D dark blue). When $\mu_{o}$ was set to 0.55 ms, CV at all firing rates increased above the CV∗ boundaries in the same way as those observed during the Cathodic Block with $\mu_{o}$ = 3 ms (Figure 3C light blue, shaded dark blue with arrow, respectively). Based on this similarity to the CV∗ relationship we hypothesize that channel density, parameterized as channel conductance, should be larger to generate APs in response to this faster EPSC arrival and comply with CV∗ performance.

As described previously, the membrane conductances affect the sensitivity to electrical stimulus. We found that increasing g_Na_ and g_KH_ together increased the firing range in response to GVS stimulation (Figures S1A and S1B). Meanwhile, increasing g_KL_ only increased the irregularity of firing in agreement with previous observations (Eatock et al., 2008; Kalluri et al., 2010; Hight and Kalluri, 2016). Thus, we kept g_KL_ = 1.1 mS/cm^2^ and scaled the conductance values for g_Na_ and g_KH_ to the upper limit of the biologically realistic conductance values (Hight and Kalluri, 2016). With higher conductance and with $\mu_{o}$ = 0.75 ms, simulated neurons exhibited a GVS-induced firing range of 0–188 sps and fr_o_ = 100.3 ± 2.4 sps (Figures 3D–3G purple). Although the maximum induced firing rate observed *in vivo* (∼250 sps) (Goldberg et al., 1984) is outside the induced firing range of this neuron, these values approach the realistic firing range of a vestibular afferent (Figure 3F purple), with CVs that remained within the CV∗ boundary (Figure 3G purple). The increase in firing rate, however, remained low at −0.32 sps/μΑ, CI [−0.35, −0.28], about six times smaller than reported values (black) of −2.01 sps/μΑ CI [−2.19, −1.89] (Figure 3E purple).

Previous experiments indicate that irregular afferents with calyces have strong “non-quantal” (NQ) effect that can increase afferent response to external current up to 4.5 times and has been reported to be a modulatory effect that increases response to GVS (Yamashita and Ohmori, 1990; Eatock and Songer, 2011). K+ accumulation in the synapse has been implicated as the source of the effect (Contini et al., 2017; Fuchs, 2017). Incorporation of the NQ effect into the model boosted the sensitivity in response to GVS presentation (−1.65 sps/μΑ CI [−1.67, −1.63]) (Figures 3D and 3E yellow). This simulated response more closely matches the experimental slope −2.01 sps/μΑ CI [−2.19, −1.89] (Figure 3E, black) while also adhering to the other experimental observations (Effects I–III) (Figures 3D–3G yellow).

### GVS stimulation maintains CV∗ by changing sampling variance of EPSCs

To understand how CV∗ is maintained during GVS stimulation, we examined the changes in induced current at the axon with changes in magnitude of GVS stimulation. GVS stimulation creates a baseline shift in membrane current that changes the size of all EPSCs at the membrane uniformly without changing EPSC timing (Figure 3B blue and orange). All EPSCs are released with timing and height defined by the hair cell and which can be captured with a set of stochastic functions that do not vary over time (Hight and Kalluri, 2016). Therefore, an increase or decrease in the number of EPSCs that become APs is equivalent to sampling this underlying distribution more (cathodic stimulation) or fewer (anodic stimulation) times. With more samples, the variance will decrease, and with fewer samples the variance will increase. In probability theory, this effect is commonly referred to as “sampling variance.” As a result, the standard deviation of firing rate will follow this trend, leading to a decrease in CV with higher induced firing rate. Because all EPSCs are drawn from the same distribution, firing regularity should follow the underlying distribution, leading to each neuron having a CV∗ that governs the relationship between CV and ISI. We further confirmed that the output from the hair cell is necessary to maintain CV∗ during GVS by performing the same experiment in an afferent without any EPSC arrival. This simulation shows extremely low, deviating CV values (Figure S2).

### Axonal response to GVS alone is insufficient to explain firing rate adaptation and rapid onset response

GVS stimulation of the axon produced no responses with a transient onset that adapts over seconds shown in Effect IV nor did it produce adaptation-to-baseline Effect V or high pass frequency response of Effect VI (Figure 1). The HK model that we modified did not possess any mechanisms with the response characteristics that could account for the adaptation durations seen in Effect IV. Further review of the literature revealed that the afferents and their calyceal endings contain Na_v_ 1.5 voltage-gated sodium channels (Hurley et al., 2006; Eatock et al., 2008) not originally included in the HK model. These voltage-gated channels have long recovery from inactivation that lasts over seconds with a double exponential response that could contribute to or explain the adaptation effect (Balbi et al., 2017). In addition, more recent examination of the NQ effect suggests that the permeation of K+ in the synaptic cleft via cyclic nucleotide-gated channels (HCN) increases sensitivity to EPSP release from the hair cell as the result of increased afferent activity. The dynamics of the NQ effect due to influx and efflux of K+ appear to match the long adaptation time course seen in the GVS firing rate response (Contini et al., 2017; Contini et al., 2020). To investigate the possibility that Effects IV, V, and VI could be attributed to axonal response to GVS, we introduced a more detailed dynamic NQ effect, HCN channel K+ current, and a Markov model of the Na_v_ 1.5 (Balbi et al., 2017) into the axonal model. We then applied steps of −30 μA cathodic GVS to examine the responses (Figure S3A). Although these responses clearly show the increase in firing rate, they fail to demonstrate the rapid increase in onset activity and the subsequent adaptation of Effects IV and V in Figure 1.

Afferents have been shown to have a natural adaptation pathway that responds to mechanical stimulation of the hair cell, resulting in adaptation in overall firing rate (Rabbitt et al., 2005; Boyle et al., 2009; Gensberger et al., 2016). The underlying mechanism is not understood (Rabbitt et al., 2005; Songer and Eatock, 2013), but the similarity of this adaptation time course to that seen in GVS step responses (Effect IV) suggests that GVS stimulation might be activating the same natural pathway (Rabbitt et al., 2005). For this reason, we hypothesized GVS stimulation must simultaneously affect the axon and this natural hair cell adaptation mechanism.

### GVS stimulation activates a natural hair cell pathway

No mechanistic model exists to explain hair cell adaptation, so we modify a state-space model (Rabbitt et al., 2005) that represents the phenomenological hair cell adaptation in firing rate in response to mechanical stimulation (see methods). The study that introduced this state-space model showed the adaptation response was a summation of a fast (η_f_) and a slow (η_s_) time constant component. It indicated that the fast component was less present in response to inhibitory stimulation than to excitatory stimuli, but it made no further prediction about the physiological mechanism of adaptation (Rabbitt et al., 2005).

In both studies we replicated *in silico*, afferents were stimulated for over 10 seconds, revealing an adaptation in which cathodic/anodic current initially causes an increase/decrease in firing rate that adapts to a baseline firing rate over the course of about ten seconds. Time courses and ratios of excitatory and inhibitory responses are comparable. In both studies, ±10 μA GVS steps were delivered (Figure 1C). Even at this low amplitude, there is shift in baseline firing rate after the strong initial response to the step is observable in the *in vivo* study but not apparent in the *in vitro* study.

**Figure 4:**
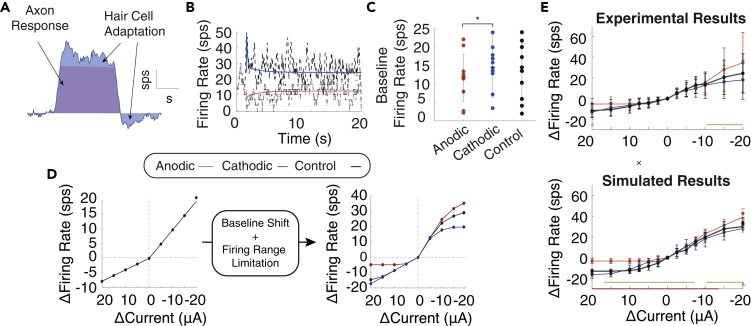
Adaptation in GVS modulated afferent response (A) The full adaptation is composed of a change in firing rate due to axonal response (purple) and hair cell adaptation, (blue) which responds to changes in internal current. (B) We can tune adaptation gains and time constants to get adaptation that resembles experimental *in vitro* results from Manca et al. (2019) to −10 μΑ of cathodic (blue) and anodic (red). (C) We find a significant baseline shift with anodic and cathodic current in the experimental results in the *in vitro* study (t(9) = 2.37, p = 0.042). (D) Without considering baseline shift and the firing range limits (*fr*_*axon*_ = 0, maximum firing rate 55 sps) the spike rate changes to current steps are predicted to be the same after baselines of anodic (red), cathodic (blue), and control or zero baseline (black) using *fr(t)* as they overlap on the plot (left). When *fr*_*axon*_*= fr*_*o*_, *fr(t)* resembles experimental results in plot (E). (E) We use *fr*_*adapt*_*(t)* to modulate *μ*_*0*_*(t)* in our full model. Traces are in the same colors. A non-parametric cluster statistic is used to compare anodic with cathodic step response (green) within conditions. The *in vitro* experimental data (above) and simulated data (below) was tested for significant differences between conditions with anodic-control (red), cathodic-control (blue), and anodic-cathodic non-parametric cluster statistic (green) shown on each image. All statistical data are presented as mean ± std.

We propose that the baseline change in firing rate is the axonal response observed earlier and that the adaptation is attributed entirely to the hair cell, as the baseline activity level increases with current amplitude with height of approximately two times the current amplitude in μA (Figure 4A and 1C black). In the *in vitro* study, we observe low overall activity, as indicated by the low spontaneous rate and lower induced firing range. We produced a similar attenuated response by reducing the conductance of the axon in the *in vitro* model such that it became less responsive to EPSCs (low fr_o_) and GVS stimulation (lower firing range) (Figure 4E black). However, because the amplitude of the instantaneous response at the onset of the GVS step (excluding the baseline) was comparable to that seen in the *in vivo* study, we predicted the hair cell adaptation pathway *in vitro* is separable for axon effects and unaffected by the change in axonal conductance. As a result, the induced firing rate over time could be represented as a function:

(Equation 1) $$fr\left(t\right)=fr_{adapt}\left(t\right)+fr_{axon}\left(t\right)$$

where *fr*_*adapt*_*(t)* is the hair cell adaptation function based on (Rabbitt et al., 2005). *fr*_*axon*_ is the axonal firing rate. Under the influence of GVS in an *in vivo* axon, *fr*_*axon*_ would be significantly different than the spontaneous rate. In the *in vitro* experiment, we assumed the axon is approximately unresponsive and *fr*_*axon*_ *= fr*_*o*_, with a maximum firing rate of 55 sps, based on the *in vivo* experimental data. So, all changes in firing rate could be attributed to hair cell response, *fr*_*adapt*_*(t)*. We used this equation to predict the contribution of the hair cell to firing rate without the influence of stochastic channel dynamics on firing rate.

Because EPSC amplitude has not been shown to vary dramatically (Dulon et al., 2009), we theorized that the mechanism by which hair cell adaptation affects axonal firing is a change in vesicle release rate by the hair cell. For simplicity, we assumed EPSC arrival rate is inversely proportional to firing rate, so we could transform the relationship between firing rate (*fr(t)*) and adaptation (*fr*_*adapt*_*(t)*) into a function for change in EPSC arrival over time (*μ(t*)) (see methods)*.* We theorized that existing hair cell pathways would need to change the rate of vesicle release in response to GVS, represented here as μ(t), and that this change could not be made instantaneously due to the complexity of protein dynamics involved in vesicle packing, release, and recycling. So, we modified *μ(t*) based on *fr*_*adapt*_*(t)* every t_d*μ*_ ms*.* We initially assumed t_d*μ*_ = *μ*_*o*_ ms. When gains and time constants of the equations for *fr*_*adapt*_*(t)* were fitted to the *in vitro* responses, the gain of the fast component was significantly larger than that of the slow component, with the time constants τ_f_ = 0.15 s and τ_s_ = 2 s. With these parameters, the model produced noisy adaptation similar to the original study (Figure 4B). However, even in the low conductance model, there was a noticeable baseline change in firing rate indicating the axon was still responding to GVS stimulation.

Effect V shown in Figure 1D and described in the original manuscript (Manca et al., 2019) suggests that the *in vitro* response to anodic and cathodic steps decays back to baseline after a prolonged 10 s step in GVS stimulation. It also shows an apparent sensitization such that after a 10 s anodic/cathodic step of stimulation the firing rate changes less to additional steps of anodic/cathodic stimulation and more to steps of the opposite polarity. We reanalyzed the original data from the *in vitro* study. As described in the publication, we confirmed that the firing rate after 10 s of cathodic or anodic baselines is not significantly different than the firing rate without stimulation (*fr*_*o*_) as was computed in the original manuscript (Manca et al., 2019). However, they are slightly, but significantly, different from one another based on our additional statistical analysis (paired t(9) = 2.37, p = 0.042) (Figure 4C). In addition, *fr*_*adapt*_*(t)* alone would predict when anodic, cathodic, and control (zero) baseline stimulation is delivered there would be no difference in response (Figure 4D left panel). Because of this we theorized that the lowered membrane conductance of the *in vitro* axon must be responsible for producing Effect V. A hyperpolarizing (anodic) step creates a new baseline closer to zero firing rate from which additional anodic steps brings the firing rate to the zero firing rate plateau. Similarly, when a depolarizing (cathodic) step is given, it creates a new baseline from which additional cathodic steps bring the axon closer to the Cathodic Block zone, again causing a plateau at the maximum firing rate. When we added the changes in firing rate from baseline offset and limited the firing rate to a maximum of 55 sps (Figure 4D right panel), changes in firing rate looked very similar to those seen experimentally (Figure 4E top panel). Finally, when we conducted the full simulation with μ(t) that varied according to hair cell adaptation dynamics, the simulated results closely matched those seen experimentally (Figure 4E). These results are consistent with the hypothesis that GVS step activates the hair cell adaptation response and modifies the baseline activity of the axon.

### Filtering effects of hair-cell-afferent model explain frequency responses

**Figure 5:**
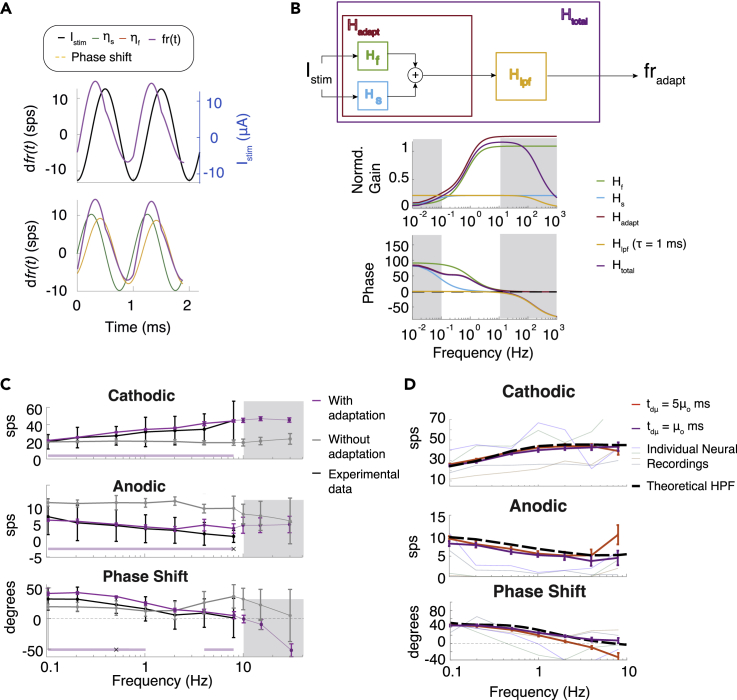
Responses to sinusoidal GVS modulation can be accounted for by the hair cell adaptation response (A) Response to sinusoidal GVS can be accounted for by the fast and the slow adaptation response of the hair cell. (B) Frequency response of *fr*_*adapt*_ comprises the fast and the slow components as well as the hypothesized low pass characteristics associated with the ability to respond to incoming EPSCs. (C) The firing rate and phase in cathodic and anodic halves of the cycle with the axon modeled with adaptation (purple) without adaptation (gray), and the original data (black). Significance of difference between with and without adaptation cases are indicated in light purple. Significance of differences between the model with adaptation and the original data are marked with x's. Data outside the original range are shown in gray. White portion correlated to frequency stimuli used in the *in vitro* experiment. (D) Examination of the low pass filtering characteristics imposed by changing the rate of EPSC sampling t_dμ_. All statistical data are presented as mean ± std.

If GVS effects on the axon are significantly attenuated in the *in vitro* study as we concluded earlier, the hair cell adaptation mechanism must be primarily responsible for the observed responses to sinusoidal GVS modulation. We investigated whether the hair cell adaptation properties described earlier could alone be responsible for the firing rate responses to sinusoids (Figure 1E). We observed that *fr*_*adapt*_*(t)* in response to 0.1 Hz sinusoids shows the phase lead (Figure 5A). On closer inspection, the hair cell adaptation response is the sum of two sinusoidal responses by the fast and slow component, and the phase lead is due to the higher-gain fast component responding more quickly to the maximum change in firing rate that occurs a quarter of a cycle (90°) before the cathodic phase of stimulation. The stochasticity of axonal firing likely smooths this response into the observed sinusoidal firing pattern observed experimentally.

The filtering effects can be broken down by performing linear systems analysis on the equations for *fr*_*adapt*_*(t)* in frequency domain*.* The fast and slow components describe two high-pass filters, H_s_ and H_f_ with corresponding cutoff frequencies of 1/τ_s_ and 1/τ_f_ (Figure 5B). We exposed the *in vitro* model to sinusoidal GVS modulation of 0.1 Hz–8 Hz, as in the study (Figure 5C white section). The simulation with the adaptation effect produced changes in cathodic and anodic firing as well as phase shifts that closely correspond to the experimental data from the *in vitro* study (Figure 5C purple and black traces). When no hair cell adaptation was included, the change in firing rate in each half of the cycle (the gain) and the phase are nearly unaffected (Figure 5C gray traces).

When we extended the analysis to higher frequencies of up to 25 Hz (gray section), the phase decreased below zero in the model including adaptation, which is not a feature of high pass filters. In addition, the decrease in phase was present at the same frequencies in the axon-only model. Because neuronal firing is limited by the timing of protein and channel dynamics, we must assume that there is a limit to how fast the neuron can respond to stimulation changes. For this reason, we incorporated a hypothetical low-pass filter H_lfp_ into the model (Figure 5B) and because we do not know the exact characteristics of this response, we assumed the cutoff to be 1kHz because the HK model could elicit 1kHz firing behavior at small values of *μ*_*0*_ seen in Figure 3A.

We considered possible mechanisms for this low pass filtering effect. One way to simulate this effect is to reduce the rate at which the EPSCs are sampled by the axon. When we slowed down the interval between samples t_d*μ*_ from $\mu_{o}$ to $5\mu_{o}$ ms, there was a decrease in firing rate in the cathodic half of the cycle and an increase in firing rate in the anodic half, consistent with a reduced gain. There is also a dip in phase below zero (Figure 5D red). These are characteristics of a low pass filter. Meanwhile at t_d*μ*_ = $\mu_{o}$ ms, the change in firing rate and phase is like a theoretical high pass filter effect (black dashed line), with phase remaining at zero (Figure 5F purple). This suggests that the update of EPSC arrival rate could be one mechanism behind the low pass filter effect. On re-examining the *in vitro* experimental data, we found trajectories of some isolated neurons (thin colored lines) were consistent with the low pass characteristics predicted by the model. This combination of high and low pass filtering effects creates a bandpass filter effect center around 1/τ_f_. The centers appear to be offset across recorded neurons, which implies vestibular afferent frequency response may be highly sensitive to small changes in head velocity frequencies in the band-pass filter range. If this theory is accurate, the frequency tuning of the irregular afferents may contribute to head velocity coding propagated to the central nervous system.

### The complete in vivo vestibular afferent model behavior predicts experimental outcomes

**Figure 6:**
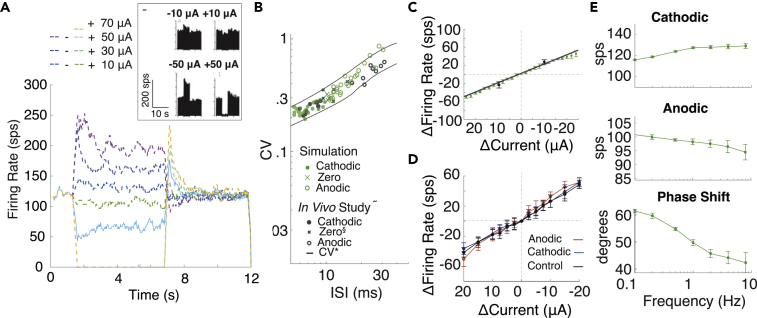
The complete effects of GVS in the *in vivo* model including hair cell adaptation (A) Firing range induced with current steps from –50 μA to 70 μA, showing adaptation and axonal response that matches *in vivo* experimental results (box). (B) The CV versus ISI associated with GVS stimulation using the model (green) compared with the CV ISI relationship in the original paper (black), which indicates cathodic stimulation (filled circle), anodic stimulation (open circle), and natural head rotation (x's). (C) The change in firing rate with cathodic current steps at slope of 2.5 sps/μA (black) as in the experimental results. (D) The change in firing rate with current steps up to ±20 from (A)10, +10, and 0 μA current baselines across five repetitions. (E) The change in firing rate to cathodic and anodic portions of sine waves of 10 μA amplitude and the phase shift to frequencies from 0.1 to 10 Hz. All statistical data are presented as mean ± std.

To create a complete *in vivo* vestibular afferent model, we combined the fitted hair cell adaptation from the *in vitro* study and previously determined *in vivo* axonal parameterization to create a complete *in vivo* afferent model. When we included both effects, the maximum induced firing rate increased to 211 ± 8.7 sps. The adaptation response found in the *in vivo* study (Goldberg et al., 1984) was closely replicated within the firing range, when we set μ_ο_ = 0.25 ms to produce fr_o_ = 120 sps (Figure 6A). In addition, when we included the adaptation effect, the change in CV versus ISI was more centered in the CV∗ lines. The percent of points within the CV∗ bounds increased from 75% without adaptation to 96.5% when adaptation was included (Figure 6B). The slope of increase in firing rate with cathodic current amplitude also increased such that it replicated the study, with a slope of −1.99 sps/μA CI [−2.03, −1.95] (Figure 6C).

We tested whether the responses to steps and sine waves change in the *in vivo* model by repeating the experiments from the *in vitro* study, because no analogous experiments were performed. We found no significant differences between responses to current steps away from the three baseline conditions (Figure 6D). This finding agrees with our theory that low membrane conductances produce these differences in the *in vitro* study (Effect V) (Figure 4); in the high conductance (*in vivo*) model, there should be no difference between these conditions, because the induced firing rate is well within the possible firing range of the neuron, so the change in amplitude remains approximately linear and not significantly different across the three conditions (Figure 6D). When we repeated the experiments that examine responses to sinusoidal stimuli, the change in firing rate during cathodic and anodic portions of each cycle had the same slope but firing rate changed around a higher starting point (Figure 6E). The phase shift had the same shape but increased by another 20°. The smaller μ_ο_ necessary to produce *in vivo* firing rates produced the overall larger phase lead (Figure S4). Together, these axonal and hair cell properties in combination can reproduce GVS effects that do not statistically differ from those observed experimentally.

## Discussion

Under the initial hypothesis that GVS stimulation affects only the axon, we started with the simplest model in the attempt to replicate all experimentally obtained results from the *in vivo* and the *in vitro* experiments (Goldberg et al., 1984; Manca et al., 2019). When the simplest model was not able to replicate all experimental data, we systematically added more biophysical features to our model until we were able to replicate all available experimental data. The mechanisms that are necessary to account for the specific aspects of afferent responses are summarized in Table 1.

### Afferent and hair cell properties implicated in GVS-modulated responses

The Hight and Kalluri (2016) model depicted vestibular firing as occurring through the axon acting as a receiver for stochastic hair cell release of vesicles. When subjected to GVS modulation, it produced CV relationships that matched natural firing properties seen in the *in vivo* experimental results. However, to accommodate the natural spontaneous activity and firing range, we had to increase the baseline EPSC arrival rate and membrane channel conductivity (corresponding to the concentration of channel expression), within physiological bounds. We also found that the sensitivity of firing rate to current amplitude was not as high as observed experimentally unless the influence of GVS on the axon was modulated by the NQ effect. This finding is consistent with past results that demonstrate the NQ effect and that it modulates external inputs to the axon (Holt et al., 2007; Songer and Eatock, 2013).

The *in vitro* experimental results showed low spontaneous activity and reduced maximal firing rate likely due to preparation effects. We can modify our model of an *in vivo* afferent to produce an accurate model of an *in vitro* vestibular afferent. We simulated the reduced axonal sensitivity (lower firing rate and induced firing range) observed in the *in vitro* experiment by reducing the membrane conductance (in our case, g_Na_) and the magnitude of the NQ effect. It is also possible to reduce sensitivity of the membrane by reducing conductivity of other membrane channels or the NQ effect in different proportions, and we make no claim as to which channels are affected by the preparation. In contrast to the axon, the hair cell appears to be unaffected by the *in vitro* preparations, which would make *in vitro* preparation an excellent paradigm for studies of hair cell properties in isolation from the axon.

Hair cell pathway being activated by GVS stimulation has been suggested in several previous studies (Kwan et al., 2019; Dlugaiczyk et al., 2019). We found that EPSC arrival rate and timing are essential to driving the spontaneous rate, inducible firing rates, and adaptation and filtering effects of a vestibular afferent. Without GVS stimulation affecting the axon, even in a low conductance axon that produces a maximum induced firing rate of only 70 sps, fast EPSC arrival rates can produce firing rates of up to 1000 sps. We believe that this indicates natural head motion is captured by change in EPSC arrival rate. Specific membrane conductances are therefore not necessarily observed in the electrophysiology experiments that are restricted to mechanical stimulation. However, replicating the experimental data from previous studies revealed the importance of specific membrane conductances in a way that had not been previously reported before this study of GVS stimulation (Goldberg et al., 1984; Smith and Goldberg, 1986). We found that an increase of both g_Na_ and g_KH_ together is required to maximize the induced firing range of a neuron with GVS stimulation (Figure S1), and we confirm g_KL_ seems to only affect firing regularity without changing induced firing rate (Goldberg et al., 1984; Eatock et al., 2008; Hight and Kalluri, 2016)

Hair cell adaptation that produces firing rate over time can be accurately simulated through an adaptation in rate of EPSC arrival from the hair cell, suggesting one mechanism of the hair cell adaptation pathway influencing axonal firing. We propose that EPSC rate remains stable without activation of the adaptation pathway and adapts when it is active. A comparison of the *in vivo* and *in vitro* response to long-term GVS steps showed that there is both an instantaneous adaptation effect and a baseline change in firing rate that increases with current amplitude and was unobservable in the *in vitro* experiment. This suggests the adaptation pathway in the hair cell is separable from the uniform increase/decrease in firing rate with cathodic/anodic current, which we deduce to be the axonal response to GVS stimulation (Figure 4A). An NQ effect is necessary to produce a large enough change in firing rate with GVS stimulation at the axon. However, the mechanism of production of the baseline shift in firing rate and of adaptation does not seem to depend on NQ effect, so we find no evidence that it is anything but modulatory on membrane current influx due to GVS stimulation as previously suggested (Fuchs, 2017).

Another important feature of the hair cell adaptation pathway appears to be that it produces a filtering effect on input signals. Our results matched those obtained experimentally in the *in vitro* experiment in which the stimulating electrodes were positioned directly in the epithelium. Because our model assumed no additional filtering due to ionic motion through tissue, we can assume that the tissue impedance did not affect the cell response in the *in vitro* experiment. We would, however, expect a frequency-dependent effect in the *in vivo* application of GVS, especially when the electrode is positioned further away from the target tissue. Tissue impedances at higher frequencies are lower than those at low frequencies due to the inverse capacitive impedance relationship with frequency (Cole, 1940). This effect decreases the sensitivity of cells to electrical stimulation at higher frequencies (>10Hz) as typified in the standard strength duration curves (Rattay et al., 2012).

Adaptation has been found to be composed of a fast component and slow component response (Rabbitt et al., 2005). We show the hair cell adaptation components create a high pass filter effect, as was observed in previous studies (Gensberger et al., 2016; Manca et al., 2019). We also find evidence of low pass filtering and propose a mechanism that could induce this effect. We theorize that each irregular vestibular afferent has a specific filtering characteristic and phase shift that only equals zero at its center frequency. As observed in a small sample of electrophysiology recordings (Figure 5F), they may have a slightly different tuning to a specific frequency of head velocity response. In this way, the irregular vestibular afferents may transfer more information about velocity through the population response than previously suggested (Sadeghi et al., 2007).

We did not have enough data on regular afferent firing to make a thorough characterization of the response to GVS stimulation. However, because regular afferents accept inputs from hair cells, they also likely have adaptation that leads to frequency-specific tuning. Similarly, axonal effects observed here should occur, but regular neurons have many fewer calyceal synapses in favor of bouton endings and therefore have a significantly reduced NQ effect, which is only seen in calyces. This likely results in the significantly lower slope of increase in firing rate with GVS current amplitude for the regular afferents as compared with irregular afferents, as previously reported (Goldberg et al., 1984). Adaptation in regular afferents has also been shown to be present but with lower amplitude than in the irregularly firing neurons, also consistent with fewer calyceal inputs in the regular afferents (Dlugaiczyk et al., 2019). We would therefore predict that regular neurons would not have the frequency-dependent phase shift in signaling observed in irregular neurons.

The mechanism of single cell firing and response to GVS stimulation has only been modeled previously by Smith and Goldberg (SG model) (Smith and Goldberg, 1986) in an attempt to explain the results obtained in the same *in vivo* study. This model can approximately produce Effects I, II, and III with the assumption that galvanic stimulation only affects the axon, but the authors note the change in firing rate does not follow the shape observed experimentally. This model predates the discovery of KL channels and the NQ effect, although the authors hypothesize differences in potassium conductances underly differences in regularity as well as that there must be a ∼4 times larger sensitivity of irregular afferents to GVS stimulation. The authors ultimately use larger magnitude EPSCs to drive irregular firing, whereas the higher density of KL channel alone can lead to dynamics that produce irregularity. In addition, we found no evidence of significant difference in EPSC amplitude between afferent types suggested by the SG model and show that only EPSC arrival rate changes are necessary to match experimental data. We note that either larger amplitude EPSCs or faster EPSC delivery would lead to more frequent summations of EPSCs as the axon would often produce a similar change in firing rate. However, our model seems to more accurately produce the effects of GVS, given our current understanding of vestibular afferent and hair cell physiology. In addition, a major contribution of this model is our ability to provide an explanation that addresses Effects IV, V, and VI, which were not explained in the SG model.

Our findings indicate that natural modulation of hair cell vesicle release rate is sufficient to explain the rapid onset followed by a slow, seconds long decay in firing rate in response to a step GVS. There are other possible mechanisms that could be involved in this response profile. (1) We investigated if axonal mechanisms alone could produce this effect, including implementing a dynamic synaptic NQ effect, HCN channel K+ currents, and Nav1.5 channel, not present in our original implementation of the HK model (Eatock et al., 2008; Contini et al., 2017). The dynamic NQ effect and both channels have long time constants on the order of the adaptation terms observed in experimental data. These effects alone could not produce transient changes in firing (Figure S3). (2) Given the presence of the dynamic NQ effect in the hair cell—afferent synapse, it may be possible that instead of GVS affecting the hair cell and producing changes in vesicle release directly, it could change the axonal firing rate, which then in turn would change the K+ concentration in the synaptic cleft, causing the hair cell to modulate its vesicle release rate with its natural inherent dynamics following the NQ mechanisms described by Contini et al. (Contini et al., 2017; Contini et al., 2020). There was insufficient data on calyceal K+ concentrations to model this effect directly. (3) Efferents have been shown to cause rapid increase in sensitivity of the afferent that decays over time (Ramakrishna et al., 2021). It is not clear, however, how this effect could explain the rapid firing rate decrease in response to the hyperpolarizing anodic step. A validated computational model of the efferent activity is not available at this time and we could not introduce it into the model. (4) Our model replicates experimental cell dynamics in form of a single afferent receiving input from a single hair cell. Rabbitt et al. (Rabbitt et al., 2005) results, used to develop this aspect of the model, show that a step cupula displacement leads to a rapid onset and subsequent decay of afferent firing rate. This implies that inputs from multiple hair cells produce the observed transient afferent firing response. We do not have data to indicate if a single hair cell or combinations of hair cells could produce the EPSC pattern that generates the transient response from the afferent.

The focus of our investigation was on understanding the axonal and hair cell components involved in GVS stimulation. To do this we modeled the axon as a single point model with equations modulating axon inputs and dynamics to account for properties of a hair cell and an afferent. This approach was established by Hight and Kalluri to successfully understand the role of channel dynamics in firing regularity. Because the anatomic features are not included in this model, it is not possible to determine the influence of electric current on the hair cell versus the axon directly using current propagation techniques. However, our models can predict the magnitude with which GVS affects membrane potential and EPSC arrival. Adding morphologic details to this model will undoubtedly provide further information on the effects of electric fields on the hair cell and the axon. We also do not exclude the possibility that these effects may reveal alternate explanations to the observed phenomena.

### Implications of targets of GVS stimulation for integration in prostheses

A limitation to producing naturalistic firing would be if the neuron has a reduced firing rate due to low EPSC arrival rate, in contrast to the low firing rate due to reduced membrane conductance implicated in the *in vitro* experiment. This effect is likely to be seen in the gentamicin treated animals (Hirvonen et al., 2005; Sultemeier and Hoffman, 2017) and are likely to occur in patients in need of the vestibular prosthesis (Aw et al., 2008). Then, GVS stimulation is more likely to elicit APs at times when no EPSCs are released, which would produce more unnatural firing statistics. GVS stimulation can induce firing rates of up to 220 sps in our simulated neuron, which approaches the maximum firing rates observed in vestibular afferents, so this limitation appears to be minimal. However, it still requires testing to determine whether the naturalistic rate and statistics of firing produced by GVS stimulation is correctly received by downstream targets of GVS stimulation. Past experiments in which hair cells were impaired on one side of the vestibular system and replaced with GVS stimulation produced VORs that more closely resembled natural eye movements than stimulation with pulses (Aplin et al., 2019a, 2019b). This appears to indicate that the GVS-evoked afferent firing patterns are well received by downstream targets and therefore useful in connecting damaged neuron in neural circuitry.

The finding that GVS stimulation affects both the axon and the hair cell in the vestibular system suggests that similar effects could in principle be advantageous in prosthetic replication of other sensory functions. (1) GVS affects end organs and smaller receptor cells rather than just axons as is the case with pulsatile stimulation. This means that in principle one could affect inputs that are further upstream in neural processing, allowing for potentially more natural responses that could engage the same molecular and cellular machinery as in the normally behaving physiological system. For cochlear implants this would mean that hair cells could be targeted rather than just spiral ganglion cells. For retinal implants it means that bipolar or photoreceptor cells could be targeted rather than the retinal ganglion cells and therefore using the natural significant processing capability of the retina. (2) GVS can induce graded amounts of excitation or inhibition through membrane potential changes that can match the natural system firing rates rather than relying on the more artificial activation of the axons with pulsatile stimulation. Meanwhile, pulsatile stimulation will have limitations on maximum firing rates that are dependent on pulse amplitude (Steinhardt and Fridman, 2020). (3) GVS can capture natural stochastic firing patterns that could be important to the system. We already know for example that in the vestibular system, pulsatile stimulation causes severe attenuation in the central nuclei in response to concerted firing evoked by pulse trains (Mitchell et al., 2017) possibly due to repeated synchronous afferent activation (McElvain et al., 2010). Similar realization in the cochlear implants has led to the development of high rate stimulation paradigms (Litvak et al., 2003), which can desynchronize pulse-evoked activity, but there is no evidence this produces firing that matches the natural stochastic patterns. GVS may be able to evoke the hair cell and axonal responses that maintain natural firing statistics.

### Limitations of the study

Although our findings strongly implicate the combined hair cell and afferent response to galvanic stimulation based on the experimental data obtained to date, we acknowledge that there may be other potential explanations that could account for the same vestibular afferent responses to galvanic stimulation.

### Resource availability

#### Lead contact

Gene Y. Fridman gfridma1@jhmi.edu.

#### Materials availability

There were no physical materials used in this computational study.

#### Data and code availability

All MATLAB code used for computational experiments described in this publication is available at the following web link along with a readme.txt file that documents the proper use of the files:

https://www.dropbox.com/sh/3re0wn2hknr2965/AAArJYB7NMrWjpLUNWr42H4wa?dl=0.

## Methods

All methods can be found in the accompanying transparent methods supplemental file.

## Supplemental information

Document S1. Transparent methods and Figures S1–S4
