# Supplementary material for: Direct current effects on afferent and hair cell to elicit natural firing patterns
Source: iScience. 2021 Feb 20;24(3):102205. doi: 10.1016/j.isci.2021.102205 (PMC7967006; doi:10.1016/j.isci.2021.102205)
Supplement: Document S1. Transparent methods and Figures S1–S4 [file mmc1.pdf]

iScience, Volume *24*

Supplemental information

Direct current effects on afferent and hair cell to elicit natural firing patterns

Cynthia R. Steinhardt and Gene Y. Fridman

Supplemental Information
Transparent Methods
Vestibular Axon Model

Vestibular afferents are categorized by firing regularity into two types: Type I (irregular) and Type II (regular) neurons. Both types of afferents differ in physiology, synaptic inputs, and channel expression. However, the Hight & Kalluri model showed that vestibular firing can be simulated accurately. Type I and Type II neurons are modeled as differing only in channel expression and EPSC magnitude (K), and interval (μ_o). The model also uses a set of non-linear differential equations to simulate channel dynamics for a number of channels specific to vestibular afferents, finding only a sodium (Na), high-voltage gated potassium (KH), and low-voltage gated potassium (KL) channel as well as a leak term are necessary to reproduce firing dynamics. The membrane potential (V) varies as:

$$\frac{dV}{dt} = 1/(C_m S)(I_{Na} + I_{KL} + I_{KH} + I_{leak} + I_{epsc} + I_{stim}) \quad (1)$$

where in addition to the current from each channel, membrane potential is influenced by the EPSCs arriving at the hair cell (I_{epsc}) and the injected current (I_{stim}). The system of equations in (Hight and Kalluri, 2016) represents each cell as a single node with overall surface area, $S = 1.1 \cdot 10^{-5}$ cm^2 and capacitance $C_m = 0.9$ μF/cm^2.

For this study, we used a modified version of the Hight & Kalluri (HK) model to simulate an irregular axon on which to test the response to GVS stimulation (Supplemental Methods). We simulated an irregular afferent, because experimental data only exist for long-term and short-term experiments on irregular neurons. Our experimental data comes from two studies (Goldberg, Smith and Fernandez, 1984; Manca *et al.*, 2019) in which the neurons have different spontaneous firing rates (100 sps and 20 sps) and different firing ranges (0-250 sps and 0-60 sps).

In (Hight and Kalluri, 2016), a range of biophysically realistic conductance values for hair cells were given: g_{Na} = 1.7-75 mS/cm^2, g_{KL} = 0-1.7 mS/cm^2, and g_{KH} = 1.8-11 mS/cm^2; these values were explored for each channel, and they found irregular firing could be imitated with conductance values: g_{Na} = 13 mS/cm^2, g_{KH} = 2.8 mS/cm^2, and g_{KL} = 1.1 mS/cm^2. In our simulations, we found g_{Na} and g_{KH} could significantly change the induced firing range of neurons, and g_{Na} had the stronger effect on firing range. Induced firing range similar to regular irregular afferents (fr = 188 sps) could be simulated with g_{Na} = 6·13 = 78 mS/cm^2, g_{KH} = 4·2.8 = 11.2 mS/cm^2, and g_{KL} = 1.1 mS/cm^2 (Supplemental Fig. S1). We construct an *in vitro* axon by lowering conductances such that the induced firing range matched that observed in the study (Manca *et al.*, 2019). There are multiple ways to model a lower conductance axon with lower firing range. To minimize changes in parameters we only decrease g_{Na} to 7.8 mS/cm^2, and we decreased μ_o to 8-15 ms, to produce lower spontaneous rate, fr_o of 15-20 sps.

In the HK model (Hight and Kalluri, 2016), hair cells are simulated with a stochastic function that releases EPSCs at a certain rate (μ_o) and with certain quanta size (K) for the whole simulation that drives the spontaneous firing rate. The authors find that a number of combinations of K and μ result in the correct firing properties and select K = 1 and μ = 3 ms as the typical settings for simulating an irregularly firing neuron. They also tested several EPSC shapes, noting shape did not have a significant effect on the range of K and μ_o used to obtain the correct firing properties. We use the EPSC shape matched synaptic current recordings from calyx terminals, with α = 0.4 for all studies. To match the spontaneous rate of each study, we assume the quanta size (K =1) is maintained across studies and set the spontaneous rates by changing EPSC release rate. For a spontaneous rate of 100-120 sps, μ_o = 0.55-0.75 ms was used. For a spontaneous rate of 15-20 sps (with the lower firing range conductance values), μ_o = 8-15 ms was used.

Only internal current stimulation is modeled in the HK study. We added external GVS stimulation to the model as a point source; the current experienced at the axon is reduced by the distance of the axon to the point source, r, which for an object x vertical and y horizontal distance from the point source is: $r = \sqrt{(x^2 + y^2)}$. Then, the current change of the axon would be the surface area of the axon (S) times the current per surface are at a distance r. The increase in firing rate with I_{axon} is significantly lower than reported in (Goldberg, Smith and Fernandez, 1984), and the original HK model does not include non-quantal (NQ) modulation. We added an NQ effect that amplifies current change, $k_{NQ} = 4.5$, and found the slope of increase in firing rate with current amplitude approaches previously reported levels.

$$I_{axon} = -k_{NQ} S \frac{I_{stim}}{4\pi r^2}$$

The lower conductance value axon must be responsive to GVS stimulation but at a lower level than *in vivo*, so, when simulating a low conductance axon, we set $k_{NQ} = 1$ to reduce responsiveness of the axon to GVS stimulation.

The channel equations used in the original model can be found below.

$$\frac{dV}{dt} = \left(\frac{1}{C_m S}\right)(-I_{KL} - I_{Na} - I_{KH} - I_{leak})$$

For every state:

$$\frac{dx}{dt} = (x_\infty - x)/\ \tau_x$$

Sodium Channels

I_{Na}

E_{Na} = 82 mV

$$I_{Na} = \boldsymbol{g}_{Na} m^3 h S(V - E_{Na})$$
$$m_\infty = \left(1 + e^{\frac{-V+38}{7}}\right)^{-1}$$

$$h_\infty = \left(1 + e^{\frac{V+65}{6}}\right)^{-1}$$
$$\tau_m = 10\left[5e^{\frac{V+60}{18}} + 36e^{\frac{-V+60}{25}}\right]^{-1} + 0.04$$
$$\tau_h = 100\left[7e^{\frac{V+60}{11}} + 10e^{\frac{-V+60}{25}}\right]^{-1} + 0.6$$

Potassium Channels

E_K = -81 mV

I_{KH}

$$\phi = 0.85$$
$$I_{KH} = \boldsymbol{g}_{KH} S(\phi n^2 + (1 - \phi)p(V - E_K)$$
$$n_\infty = \left(1 + e^{\frac{-V+15}{5}}\right)^{-0.5}$$
$$p_\infty = \left(1 + e^{(-V+23)/6}\right)^{-1}$$
$$\tau_n = 100\left[11e^{(V+60)/24} + 21e^{-(V+60)/23}\right]^{-1} + 0.7$$
$$\tau_p = 100\left[4e^{(V+60)/32} + 5e^{-(V+60)/22}\right]^{-1} + 5$$

I_{KL}

$$I_{KL} = \boldsymbol{g}_{KL} S w^4 z(V - E_K)$$
$$w_\infty = \left(1 + e^{\frac{-V+44}{8.4}}\right)^{-\frac{1}{4}}$$
$$z_\infty = (1 - \gamma)\left(1 + e^{\frac{V+71}{10}}\right)^{-1} + \gamma, \gamma = .5$$
$$\tau_w = 100\left(6e^{\frac{V+60}{6}} + 16\left(\mathrm{e}^{-\frac{V+60}{45}}\right)\right)^{-1} + 1.5$$
$$\tau_z = 1000\left(e^{\frac{V+60}{20}} + 16e^{\frac{-V+60}{8}}\right)^{-1} + 50$$

E_{leak} = -65 mV

$$I_{leak} = g_{leak} S(V - E_{leak})$$

Additionally, in supplemental work, we assessed the contribution of other axonal channels to firing to determine whether the axon alone could produce the transient effect. The equations used for this analysis can be found below.

Other Channels Added into the model:

<u>HCN a.k.a. I_h</u>

$$I_h = g_h(1 - r^3)S(V - E_h)$$

$$r_\infty = \left(1 + e^{\frac{-V+100}{7}}\right)^{-1}$$

$$\tau_r = 10^5\left(237e^{\frac{V+60}{12}} + 17e^{\frac{-V+60}{14}}\right)^{-1} + 25$$

<u>$I_{Nav1.5}$</u>

A Markov model implementation of a Nav1.5 channel opening and closing was modified into an efficient matrix multiplication in MatLAB. The original code as individual equations can be found in Balbi et al. (Balbi, Massobrio and Hellgren Kotaleski, 2017). This model relies on capturing changes between two closed states, two open states, and two inactivated states. The rows of the matrix were in the order B (magnitude), v (hemiactivation voltage), k (slope factor). This matrix *Y* was 12 x 6. The transitions were separated into a hyperpolarizing and a depolarizing component with the same three variables:

State Transitions	$\mathbf{B_{hyp}}$	$\mathbf{v_{hyp}}$	$\mathbf{k_{hyp}}$	$\mathbf{B_{dep}}$	$\mathbf{v_{dep}}$	$\mathbf{k_{dep}}$
**C1C2**	0	0	0	10	-13	10
**C2C1**	1	-43	8	10	-13	-10
**C201**	0	0	0	10	-23	-10
**O1C2**	1	-53	8	10	-23	-10
**C2O2**	0	0	0	0.05	-10	-10
**O2C2**	2	-50	10	0.05	-10	-10
**O1I1**	7	-44	13	10	-19	-13
**I1O1**	0.00001	-20	10	0	0	0
**I1C1**	0.19	-100	7	0	0	0
**C1I1**	0	0	0	0.016	-92	-6
**I1I2**	0	0	0	0.00022	-50	-5
**I2I1**	0.0018	-90	30	0	0	0

The states were a vector in the order:

A, the transition rates were calculated as follows for all state transitions simultaneously, producing a 12x1 vector:

$$A = Y_1\left(1 + e^{\frac{V-Y_2}{Y_3}}\right)^{-1} + Y_4\left(1 + e^{\frac{V-Y_5}{Y_6}}\right)^{-1}$$

A then needed to be multiplied by the current states to get the correct transition probabilities over time. The states were arranged in the vector *x* in the order (O1, O2, C1, C2, I1, I2).

The transitions in and out of state were then calculated in a matrix form with the following equation *M*:

$$\begin{vmatrix} -(A_4 + A_7) & 0 & 0 & A_3 & A_8 & 0 \\ 0 & -A_6 & 0 & A_5 & 0 & 0 \\ 0 & 0 & -(A_1 + A_{10}) & A_2 & A_9 & 0 \\ A_4 & A_6 & A_1 & -(A_2 + A_3 + A_5) & 0 & 0 \\ A_7 & 0 & A_{10} & 0 & -(A_9 + A_{11} + A_8) & A_{12} \\ 0 & 0 & 0 & 0 & A_{11} & -A_{12} \end{vmatrix}$$

$$x(t+1) = dtM + x(t)$$

$$I_{Na} = \boldsymbol{g}_{Na}S(x_1 + x_2)(V - V_{Na})$$

Where $x_1 + x_2$ is the total probability of being in the open state. *S* is the same surface area of the axon used above. *dt* was the same as for the rest of the study (1e-3 ms).

Dynamic NQ effect:
There were no existing data that could be used to model the mechanism of K+ concentration change in the synaptic calyceal cleft and the resulting effect on the axon and hair cell. Instead, we created a more realistic phenomenological model of the non-quantal effect based on trajectories of the non-quantal effect in response to current and voltage steps (Contini, Price and Art, 2017).

$\tau_{NQ} = 100\,ms$, $g_{NQ} = 0.025$ to get the correct trajectory and the change in current necessary to produce the size of non-quantal effect needed to replicate the slope of change in firing rate with DC stimulation. These equations transform internal DC current (*s*) at the afferent into the current at the axon with the non-quantal effect trajectories of slow rise with current amplitude (*n*).

$$\frac{dn}{dt}(t) = g_{NQ}s(t) - \frac{1}{\tau_{NQ}}n(t-1)$$
$$n(t) = n(t-1) + \frac{dn}{dt}(t)$$

Then, *n* can be directly added to received EPSC inputs to produce the change in internal current over time at the axon.

Hair Cell Adaptation Effect

In the *in vitro* experiments (Manca *et al.*, 2019), an adaptation in response to a 10 s GVS current step, decayed with time constants of up to 8.5 secs. In the literature, an adaptation of time of up to 13 secs in firing rate was found to natural, mechanical stimulation (Rabbitt *et al.*, 2005) . We hypothesize that GVS stimulation can activate this natural adaptation mechanism in the hair cell, resulting in the observed adaptation in Manca *et al.* (2019). In Rabbitt, *et al.* (Rabbitt *et al.*, 2005), the adaptation was shown to have two components, represented as two hidden states, a slow state and a fast state. Both states have the same state evolution equations

$$\frac{d\eta_k}{dt} = g_k\frac{ds}{dt} + \frac{g_{\infty k}}{\tau_k}s - \frac{1}{\tau_k}\eta_k$$

, where *s* is the stimulus signal, $g_o$is the instantaneous gain to a change in the signal, and g_∞ is the steady state gain to which the signal will adapt (Rabbitt *et al.*, 2005). We hypothesize that the g_∞ term, a baseline shift in firing rate is negligible compared to larger axonal effects. So, we set g_∞ = 0, such that $\frac{d\eta_k}{dt} = g_k\frac{ds}{dt} - \frac{1}{\tau_k}\eta_k$ for both adaptation states.

The total adaptation in firing rate (fr_{adapt}) is a sum of the two states we call *s* and *f*, where the response to excitatory mechanical stimulation has a larger fast component than the response to inhibitory stimulation:

$$fr_{adapt} = \eta_s + r\eta_f, \qquad r = \begin{cases} \alpha & \eta_f < 0 \\ 1 & \eta_f \geq 0 \end{cases}$$

The choice of α was not carefully measured in the paper; we set α = 0.1. For a different choice of α, g_s and g_f would have to be adjusted to fit the data.

The hair cell affects firing rate in the HK model through the stochastic process that generates EPSCs with a magnitude (*K*) and inter-EPSC timing (μ). We theorize μ(t) is a function of fr_{adapt}, because hair cells do not typically modify the packing of vesicles (*K*), but vesicle release rates have been shown to change in response to stimuli (Dulon *et al.*, 2009). Adaptation was modeled as an additive effect on top of the natural firing rate, so that $fr(t) = fr_{axon} + fr_{adapt}(t)$, due to evidence of a separable hair cell adaptation pathway and axonal responsiveness to GVS stimulation. In this absence of stimulation fr_{axon} would just be fr_o. We assume firing rate and EPSC release rate are approximately linearly related, such that $fr_o = \frac{k_{fr}}{\mu_o}$. Then, the *fr* relation can be solved in terms $\mu(t)$:

$$\frac{k_{fr}}{\mu(t)} = \frac{k_{fr}}{\mu_o} + fr_{adapt}(\text{t})$$
$$\mu(t) = \frac{\mu_o}{1 + \frac{\mu_o}{k_{fr}} fr_{adapt}} = \frac{\mu_o}{1 + \frac{fr_{adapt}}{fr_o}}$$

EPSC generation in the HK model is performed in windows, while $fr_{adapt}(t)$ is a continuous function. We assume EPSC delivery is a discretized process and therefore changes in release rate would not be immediate. We divided the trial into windows of length $t_{d\mu} = \mu_o$ ms, and in each window the stochastic EPSC generation equation was used to generate μ_o ms of EPSC trains which were concatenated to create the final EPSC train used during simulation experiments. μ_o ms was chosen because with $\mu(t)$ updating every μ_o ms EPSC summation to faster or slower EPSC releases rates would be uneffected, and at this value phase shift and firing rate results replicate the sine wave experiment results.

Next, we tuned the equation to the observations from Manca *et al.* (2019) to select values of g_s and g_f. We model GVS stimulation by setting $s = -I_{stim}$ with the current amplitude in μA, because negative/positive current causes an excitatory/inhibitory effect on firing. We fit the initial change in firing rate to the current step in (Manca *et al.*, 2019) such that

$$fr_{adapt} = \begin{cases} \left(g_s + 0.1g_f\right)\frac{ds}{dt} & \eta_f < 0 \\ \left(g_s + g_f\right)\frac{ds}{dt} & \eta_f \geq 0 \end{cases}$$

for $\frac{ds}{dt} = 10$ and $\frac{ds}{dt} = -10$, finding $g_s = 0.375$ and $g_f = 2.25$. We find the time constants associated with each component by initially assuming the median time of adaptation to anodic and cathodic stimulation ($\tau_- = 0.99\, s$ and $\tau_+ = .53$ s) are the weighted sums of the time constants, weighted by g_s and g_f. This would result in $\tau_s = 2.26\, s$ and $\tau_f = 0.24$ s. τ_f and τ_s control the frequency at which the phase shift goes to zero. We decrease $\tau_f = 0.15$ s and $\tau_s = 2$ s to better replicate experimental results. This is a minor change within the biophysical range (Rabbitt *et al.*, 2005). We also find that when the $\mu(t)$ function is used to change EPSC release rate in the full simulation, results better match the experiment when gains are amplified two-fold to $g_s = 0.75$ and $g_f = 4.5$.

Simulating Firing Regularity Experiments
The change in firing rate with GVS stimulation amplitude was measured by applying one-second GVS stimulation fields at each current amplitude between -100 μA to 70 μA. In each trial, stimulation steps were preceded with a 50 ms window without stimulation to assure the membrane potential was at rest. APs in this time window were excluded. A trial with 1050 ms of stimulation at each current amplitude was performed nineteen times with different random seeds to replicate experimental results (Goldberg, Smith and Fernandez, 1984); the CV versus ISI comparison, the change in slope, and the maximum firing rate across neurons were found across the population. The action potentials were detected from the voltage trace by finding points where the voltage was above -35 V and greater than the voltage 0.01 ms before and after. The CV and ISI were calculated from the detected times of action potential peak. The slope of increase with cathodic current was found by only including current amplitudes less than 0 μA and which produced an increase in firing rate compared to lower amplitude cathodic current stimulation. The trend of increase was fit with fifth-order polynomial, and the last current for which the curve had positive slope was the lowest current amplitude point included.

Simulating Adaptation Experiments
Adaptation was captured in both replicated experiments but had a different response. We hypothesized that there are two components to the responses to GVS stimulation, an axonal response that produces an overall change in in firing rate while current is being driven through the axon and a hair cell response that is responsible for the adaptation shape. In (Manca *et al.*, 2019), we believed the *in vitro* prep left the axon significantly less responsive than an *in vivo* axon to GVS stimulation, leading to the lower spontaneous range and smaller induced firing range. We fit the adaptation function to these data (see above). We then tested adaptation was properly captured by assuring that the response to a 10-second GVS field of +10 μA

and -10 μA produced the same initial change in firing rate with a current step and decayed back to baseline after 10 seconds.

In the *in vivo* axon model with higher conductance values and a larger NQ effect, both the NQ and adaptation effect need to be slightly reduced to replicate experimental results. We used k_{NQ} = 3.5 and scale adaptation parameters down to $g_s = 0.49$ and $g_f = 2.9$, so the ratio of g_s and g_f remain fixed. This is due to the axon being more responsive to inputs when conductances are larger. With these parameters, we confirmed that adaptation to a five-second GVS step followed by five-seconds after the current stepped down produced a change in baseline firing rate with an adaptation effect on top during the step and adaptation in the opposite direction, after it stepped down. The experiment was simulated to steps of GVS current of -50, -30, -10, 10, 30, 50, and 70 μA. The response to -70 μA could not be replicated because it induced firing rates out of the induced firing rate of our model, producing cathodic block.

Simulating Baseline Current Step Experiments

In the *in vitro* study (Manca *et al.*, 2019), a baseline of -10 μA, 0 μA or +10 μA GVS current was delivered for ten seconds then current step of ±20, ±15, ±10, ± 7.5, ±5, ± 2.5, and 0 μA away from this baseline were delivered for an additional two seconds. Changing in firing rate was compared between the baseline firing rate in the last one second of baseline and the first 50-500 ms after the current step. We repeated this experiment on ten neurons *in silico* to match the size of the study in the experimental data being replicated.

Simulating Sinusoidal Waveform Experiments

In the *in vitro* study (Manca *et al.*, 2019), the response to fifteen cycles of sinusoidal waves of height ±10 μA at frequencies of 0.1, 0.2, 0.5, 1, 2, 4, and 8 Hz was recorded across ten neurons. As in the study, we needed to determine the phase shift of the response to each signal. For each simulated neuron, the phase shift was determined by fitting a sinewave of the stimulation frequency to all cycles of response to the sinewave. For lower frequencies, less cycles are required to capture the phase shift, so, for frequencies less than 1 Hz, we simulate five cycles and, for frequencies of 1 or more Hz, seventeen cycles, excluding the first and last cycle from analyses. We then measure the firing rate in 180-degree windows centered around the cathodic half of the response and anodic half of the response in each cycle and take the average. We report the firing rates per cathodic and anodic half of the response and phase shift across neurons. We extend the study to see the responses to frequencies from 0.005 Hz to 150 Hz to capture the full frequency response of the neurons. We analyze the results on *fr(t)*, which represents the change that hair cell adaptation contributes to the neural response without the noise of axonal response, and on the full axon model.

Statistical Comparison to Experimental Results

To compare slope of increase with cathodic current across models, we fit the original data(Goldberg, Smith and Fernandez, 1984) with a line of best fit with intercept zero. We compare this slope and 95% confidence interval of fit to the slope and 95% confidence interval of fit of each of our models. If the experimental slope is within the bounds of the model, we declare the experimental data replicated.

To compare CV-ISI relationships between the experimental study and the simulated results, we use the equation for CV* with values that match the 95% confidence interval observed in the study. We then count the number of points between 5 ms and 50 ms that fall within these bound, checking whether over 95% fall within experiment the 95% confidence bounds.

To compare the change in firing rate with current steps from three baseline conditions and change in firing rate and phase with sinewave frequency, we perform a non-parametric cluster statistic at the level of $p < 0.05$. Between conditions we have two groups of neuronal responses. We permute neuron identity groups 500 times and find clusters of values that significantly differ from permutation results and reality. The t-value of the cluster needed to exceed 3 to be significant. We used this test for comparisons within experimental (Manca *et al.*, 2019) and simulated data between baseline conditions. We also compare results of the sinewave experiment with and without adaptation. We also compare experimental and simulated results of both experiments.

Supplemental Methods

Modification to Channel Dynamics for Long-term Stability

The dynamics of the high-voltage gated potassium (KH) channel were changed such that KH channels were slower and sustained firing for up to several seconds without showing instability; these values more closely matched previous models of KH channels (Rothman and Manis, 2003). I_{KH}, the current from the KH channel, is equal to the total conductance across the surface area of the node of Ranvier ($\overline{g_{KH}}\, S$) times the probability of the channel being activated (*n*) or inactivated (*p*) times the difference between V and the reversal potential of potassium (E_k); the probability of KH channels being activated was $\phi = 0.85$:

$$I_{KH} = \overline{g_{KH}}\, S[\phi n^2 + (1-\phi)p](V - E_K) \qquad (2)$$

The inactivation/activation variable x (i.e. n and p) has rate of change ($\dot{x}$) defined by

$$\dot{x} = x_\infty - x/\tau_x \qquad (3)$$

where τ_x is the time constant of *x*, and x_∞ is the steady-state value. For the KH channel, all dynamics were kept the same as (Hight and Kalluri, 2016) except the time coinstant for *p* was reverted to the equation from (Rothman and Manis, 2003).

$$\tau_p = 100\left[4e^{\frac{V+60}{32}} + 5e^{-\frac{V+60}{22}}\right]^{-1} + 5 \qquad (4)$$

Supplemental Figures

Supplemental Figure S1 associated with axonal conductances investigation in Figure 3**. a)** Increasing g_{Na}, g_{KH} and g_{KL} from initial value to highest value possible within biologically realistic values. Original values (black) compared to increase. **b)** Result of increasing g_{Na} in combination with each increase in g_{KH} at each tested value of g_{KH} from 2.8 to 11.2 mS/cm^2. **c)** g_{KL} effect on action potentials.

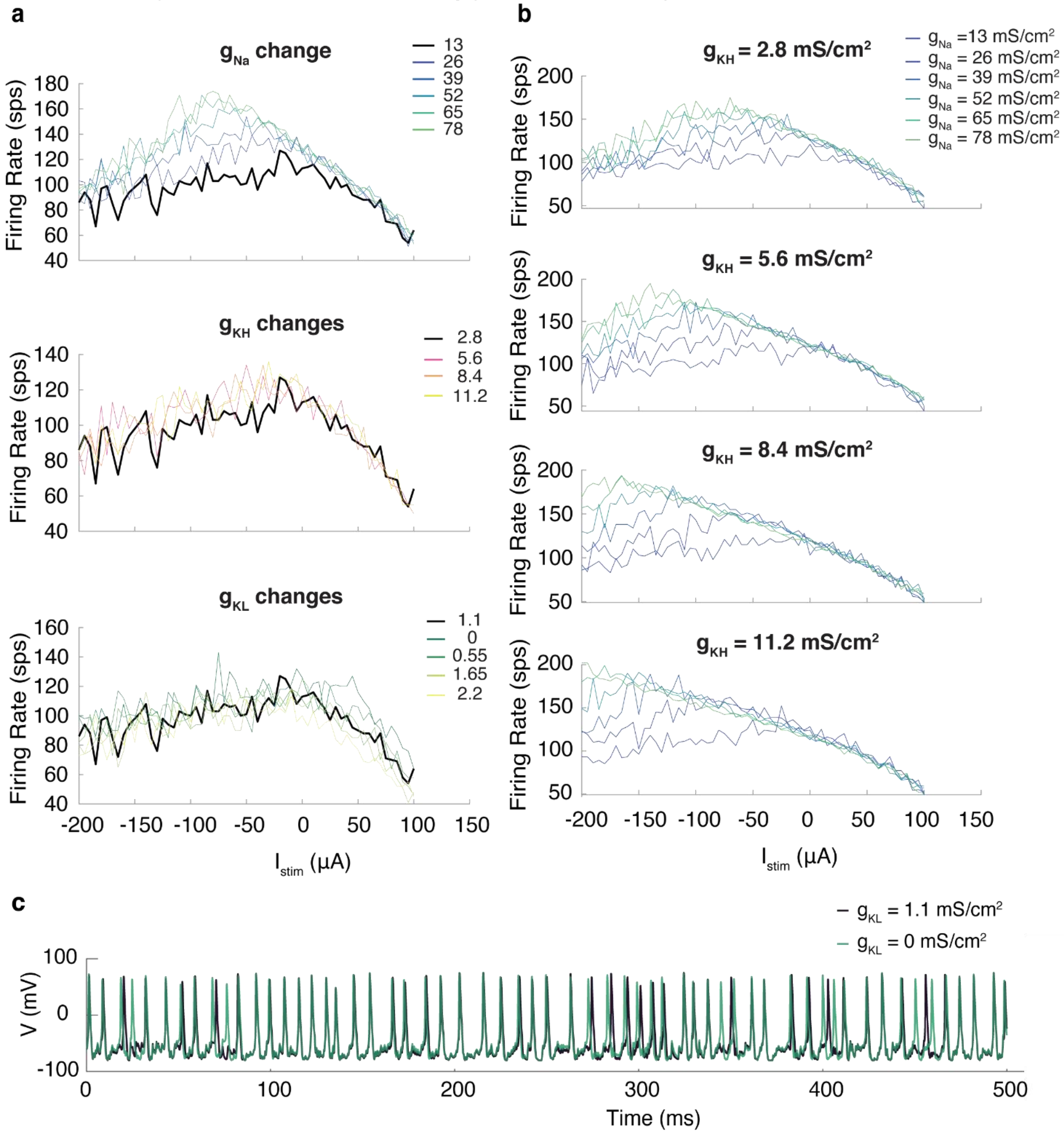

Supplemental Figure S2 associated with CV* description in Figure 3b. Support for the idea that the output from the hair cell is necessary to maintain the CV* relationship when GVS is applied. **a)** Simulated paradigm in which GVS current with amplitudes between +70 μA to -100 μA is introduced to the axon with no EPSCs **b)** The CV vs ISI relationship in this case produces much lower CVs than the CV* found in experimental data. **c)** The induced firing range is approximately the same but firing rate increases from 0 sps at 0 μA of stimulation to the maximum firing rate.

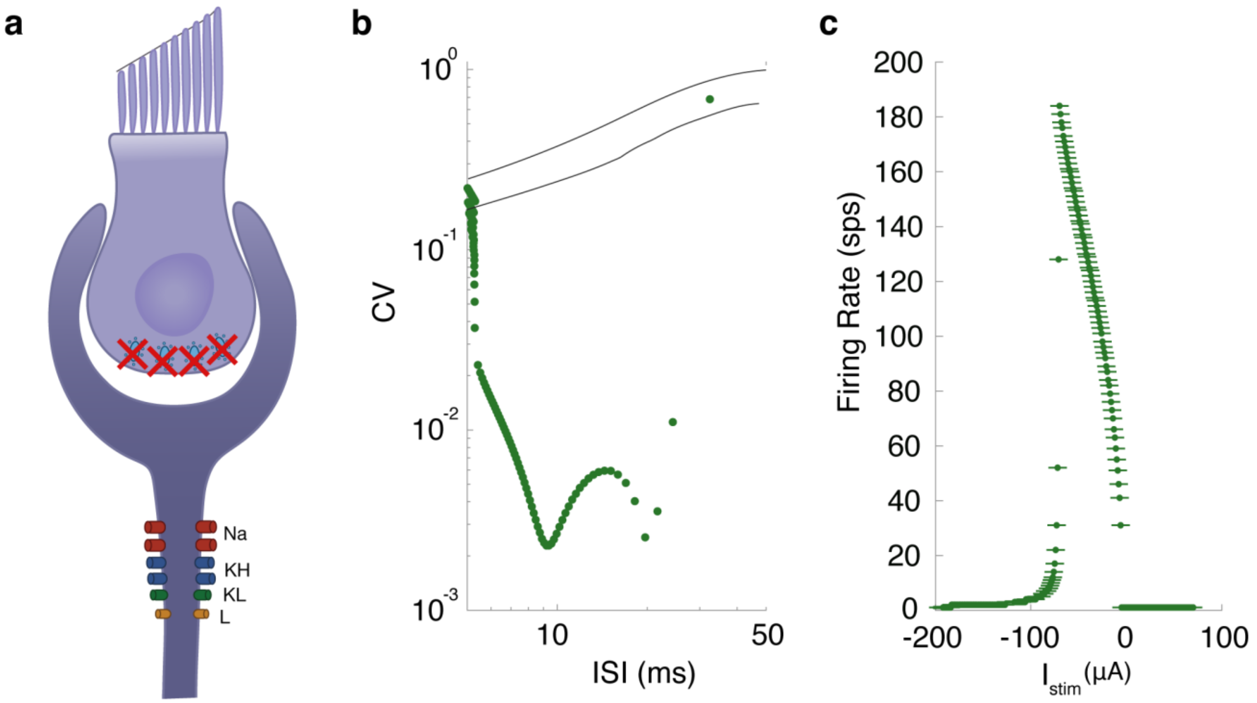

Supplemental Figure S3 shows the effect of inclusion of Na$_v$ 1.5, HCN channels and Dynamic NQ response is insufficient to explain rapid onset and adaptation effects IV and V discussed in Figure 1. **a)** Multiple manipulation of conductances and introduction of Na$_v$ 1.5, HCN, and Dynamic NQ response *without* hair cell GVS response fails to show the rapid onset followed by slow adaptation seen in the experimental data. **b)** For the complete axon *with* hair cell simulation, Dynamic NQ effect that mimics the dynamics of the influx and efflux of K+ into the synaptic cleft has only minor impact on step responses over the constant Scalar NQ effect (colored lines are anodic and cathodic steps in μA).

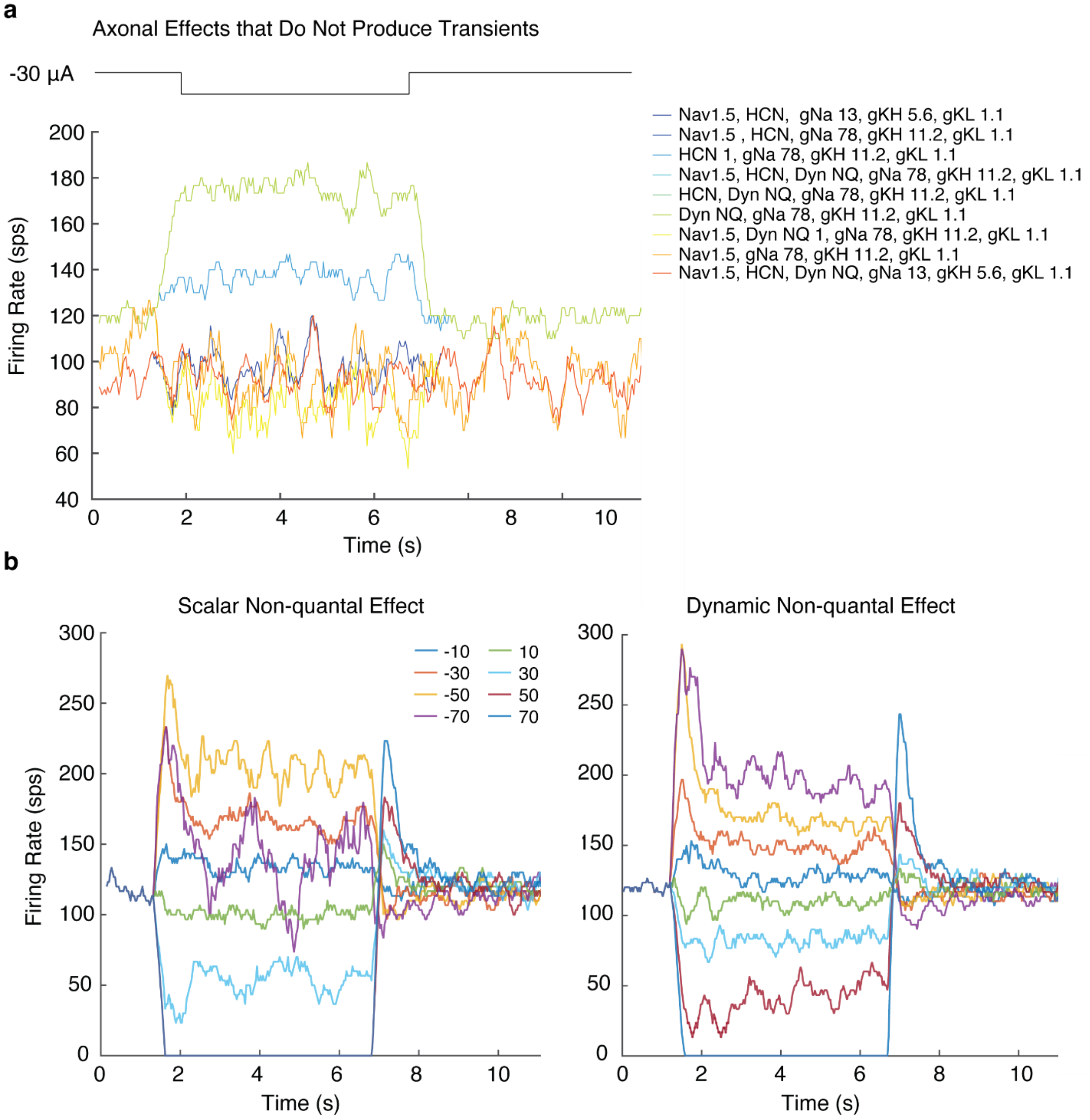

Supplemental Figure S4 shows the smaller μ_o necessary to produce *in vivo* firing rates, produces the overall larger phase lead discussed in Figure 6.

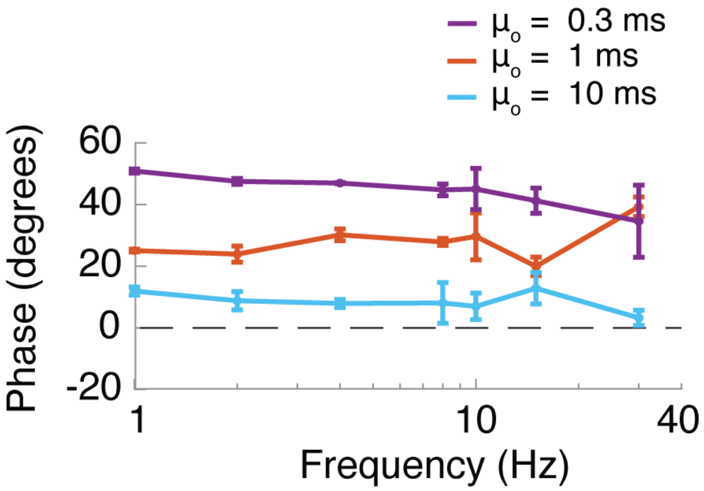

Supplemental Reference

Rothman, J. S. and Manis, P. B. (2003) 'The roles potassium currents play in regulating the electrical activity of ventral cochlear nucleus neurons', *Journal of Neurophysiology*, 89(6), pp. 3097–3113. doi: 10.1152/jn.00127.2002.
